# ﻿New contribution to the leafhopper genus *Thapaia* (Hemiptera, Cicadellidae, Typhlocybinae), with the description of a new species from China

**DOI:** 10.3897/zookeys.1254.155062

**Published:** 2025-10-01

**Authors:** Xiaojuan Yuan, Wenming Xu, Zihui Zhao, Di Su, Yuehua Song

**Affiliations:** 1 School of Karst Science, Guizhou Normal University, Guiyang, 550025, China Guizhou Normal University Guiyang China; 2 State Engineering Technology Institute for Karst Desertification Control, Guiyang, 550025, China State Engineering Technology Institute for Karst Desertification Control Guiyang China

**Keywords:** Erythroneurini, morphology, taxonomy

## Abstract

*Thapaia malingensis* Yuan & Song, **sp. nov.**, a new species of Erythroneurini (Hemiptera, Cicadellidae, Typhlocybinae) from Maling River Canyon in Xingyi, China, is described and illustrated. A revised description of the genus is also provided, and a key to males of all species worldwide is given.

## ﻿Introduction

Erythroneurini is the largest tribe within the subfamily Typhlocybinae (Young 1952). According to Dmitriev et al. (2022), approximately 2045 species in about 210 genera have been documented worldwide (Dmitriev et al. 2022). The ecological significance of the Erythroneurini lies in its dual impacts on agricultural ecosystems and natural balance. As a phytophagous group, most species contribute to material cycles and energy flows by feeding on plant leaves and shoots, thus serving as key subjects for research on insect–plant coevolution. However, certain species pose a threat to agriculture due to their specific niche traits. *Elbelus tripunctatus* (Hemiptera, Cicadellidae, Erythroneurini) feeds on crops such as rice and maize with its piercing-sucking mouthparts, causing leaf chlorosis, stunted growth, and significant yield losses (Cao 2014; Yuan et al. 2025). Additionally, a few species, upon being artificially introduced to new regions, spread rapidly in the absence of natural enemies. These invasive species not only outcompete local insects for habitat but also exacerbate ecological risks by acting as virus vectors, thereby challenging the stability of agroforestry ecosystems (Cao 2014; Yuan et al. 2025). Erythroneurini are mainly concentrated in mountainous regions and subtropical–temperate transition zones across the Oriental and Palaearctic Realms, but particularly concentrated in China and Southeast Asia. These regions, which are characterized by complex terrain (e.g. the Hengduan Mountains) and diverse microclimates, harbor extremely high species richness, driven by their environmental heterogeneity. Meanwhile, owing to the lack of systematic surveys in remote regions, numerous undiscovered species remain, offering extensive exploration opportunities for research in taxonomy, biogeography, and ecology—highlighting significant research value.

The leafhopper genus *Thapaia* Dmitriev & Dietrich, 2006, a replacement name for the junior homonym *Masaakia* Thapa, 1989 (Dmitriev and Dietrich 2006) belongs to the tribe Erythroneurini of Typhlocybinae with *Masaakia nema* Thapa, 1989 as its type species (Thapa 1989; Song and Li 2009). In 1994, the second species of the genus, namely *T. sikkimensis*, was reported by Dworakowska (Dworakowska 1994; Sohi 1998). In 2009, Song and Li (2009) reported three new species: *T. multibudna* Song & Li, 2009, *T. plumula* Song & Li, 2009, and *T. tina* Song & Li, 2009. Cao et al. (2019) described a new species, *T. tibetensis* Cao, Dmitriev, Dietrich & Zhang, 2019, and redefined and described the genus in terms of *T. plumula*. Here, we summarize the seven species of this genus. Among them is a new species, *T. malingensis* Yuan & Song, sp. nov., from the karst area of southwestern China.

## ﻿Materials and methods

All leafhopper specimens were collected and preserved in absolute ethanol. The morphological terminology used in this study follows the definitions provided by Dietrich (2005), Song and Li (2009), and Cao et al. (2019). The body length is measured from the apex of the vertex to the tip of the forewings. The abdomens of the specimens are removed and cleared by heating them in a 10% NaOH for 1–2 mins, then rinsed with water and stored in glycerin. To prevent the components from drying out, genitalia are dissected in glycerin. The dissected male genitalia and wings are observed and drawn using Olympus SZX16 and BX53 microscopes. The habitus images of the specimens are taken with a Keyence VHX-5000 digital microscope. Multiple images are focus-stacked to obtain focused images, and these were edited using Adobe Photoshop CS6. The holotype and other specimens of the new species are deposited at the School of Karst Science, Guizhou Normal University, Guiyang, China (**GZNU**).

## ﻿Taxonomy

### ﻿ Thapaia

Taxon classificationAnimaliaHemipteraCicadellidae

Dmitriev & Dietrich, 2006

49CEBF74-BA6C-589C-B8BD-528047F2F6B9

Masaakia Thapa, 1989: 120; preoccupied by Masaakia Takeuchi, 1950 (Hymenoptera, Tenthredinidae).
Thapaia Dmitriev & Dietrich, 2006: 37 (new name for Masaakia Thapa, 1989); Song and Li 2009: 62 (redescription).

#### Type species.

*Masaakia nema* Thapa, 1989, by original designation.

#### Amended description.

Generic characteristics according to Thapa (1989), Song and Li (2009), and Cao et al. (2019).

Body yellow or white, with dorsal patterns usually in red, orange, or brown. Head with anterior margin medially produced, slightly narrower than pronotum, or subequal to or broader than pronotum. Known species of this genus with vertex bearing a pair of dark preapical spots and a pair of dark brown spots arising downward from posterior margin; these serve as taxonomic diagnostic characters. Coronal suture distinct or indistinct. Junction of vertex and pronotum dark-colored. Face broadened, with anteclypeus almost black or brownish black, nearly pentagonal. Eyes black. Pronotum broad, often with obvious dark patterns. Scutellum yellow or orange, with basal triangles and apex black; transverse impression distinct, with two small, rounded spots above it. Forewing with large, dark patterns in bright ochre-yellow or dirty-brown, with special venation for Erythroneurini, CuA′ fused with MP proximal to MP bifurcation, leading to petiolate shape of second apical cell; MP″+CuA′ curved basally; AA fused with AP. Hind wing with RA absent.

Abdominal apodemes not reaching or exceeding 3^rd^ sternite.

**Male genitalia.** Anal tube often with pair of basal processes, distal area near ventral side with good sclerotization. Pygofer with dorsal appendages, firmly fused to the pygofer lobe at basal margins. Pygofer lobe hind margin acutely produced, often with small group of microsetae around it; several long and rigid setae, or long and slender cilia, scattered on surface of pygofer lobe. Subgenital plate expanded at base, with 4 or 5 macrosetae on lateral surface, apex bending towards internal margin, dentiform, or rounded without curve. Style apex slender or with a second end point, preapical lobe small but prominent or broad and distinct. Aedeagal shaft tubular; preatrium length variable, with or without processes; dorsal apodeme swollen slightly. Connective with distinctly developed lateral arms, central lobe distinct or vestigial, stem short or absent.

#### Distribution.

Oriental and eastern Palaearctic Regions: China (Henan, Guizhou, Sichuan, Tibet), India (Sikkim), and Nepal (Kathmandu).

##### ﻿Checklist of species of the genus *Thapaia* Dmitriev & Dietrich, 2006

***Thapaia malingensis* Yuan & Song, sp. nov.**

**Distribution.** China (Guizhou).

***Thapaia multibudna* Song & Li, 2009**

*Thapaia multibudna* Song & Li, 2009: 65; Song and Li 2014: 191.

**Distribution.** China (Guizhou).

***Thapaia nema* (Thapa, 1989)**

*Masaakia nema* Thapa, 1989: 42, 121; *Thapaia nema* (Thapa, 1989) in Dmitriev and Dietrich 2006: 37.

**Distribution.** Nepal (Kathmandu).

***Thapaia plumula* Song & Li, 2009**

*Thapaia plumula* Song & Li, 2009: 65; Song and Li 2014: 192; Cao et al. 2019: 198.

**Distribution.** China (Henan, Sichuan).

***Thapaia sikkimensis* (Dworakowska, 1994)**

*Sandanella sikkimensis* Dworakowska, 1994: 55, 124, 187; *Masaakia sikkimensis* (Dworakowska, 1994) in Sohi 1998: 93.

**Distribution.** India (Sikkim).

***Thapaia tibetensis* Cao, Dmitriev, Dietrich & Zhang, 2019**

*Thapaia tibetensis*Cao et al., 2019: 198.

**Distribution.** China (Tibet).

***Thapaia tina* Song & Li, 2009**

*Thapaia tina* Song & Li, 2009: 63; Song and Li 2014: 193.

**Distribution.** China (Henan).

##### ﻿Key to males of *Thapaia* worldwide

**Table:** 

1	Aedeagal shaft curved dorsally, without any processes	***T. malingensis* sp. nov.**
–	Aedeagal shaft with processes (apical, median, or basal processes)	**2**
2	Aedeagal shaft slender, with a pair of long apical processes (approximately 1/3 length of aedeagal shaft)	***T. tibetensis***
–	Aedeagal shaft short and thick or of medium thickness, with paired short processes	**3**
3	Aedeagal shaft without obvious large ventral process at base	**4**
–	Aedeagal shaft with one large ventral process at base	**5**
4	Paired apical processes of aedeagal shaft bifurcated	***T. sikkimensis***
–	Paired apical processes of aedeagal shaft unbranched, with a pair of subapical accessory processes	***T. plumula***
5	Aedeagal shaft with two pairs of apical processes	***T. tina***
–	Aedeagal shaft with only one pair of apical processes	**6**
6	Aedeagal shaft with one pair of small lateral processes near base	***T. multibudna***
–	Aedeagal shaft without small lateral processes near base	***T. nema***

### ﻿ Thapaia malingensis

Taxon classificationAnimaliaHemipteraCicadellidae

Yuan & Song sp. nov.

67CCBCDE-5345-5D74-86D4-8863B58C5EA8

https://zoobank.org/1D599258-6BA6-4554-8D72-BA7D87AD0DCA

Figs 1A−H, 2A−M

#### Description.

**Figure 1.**
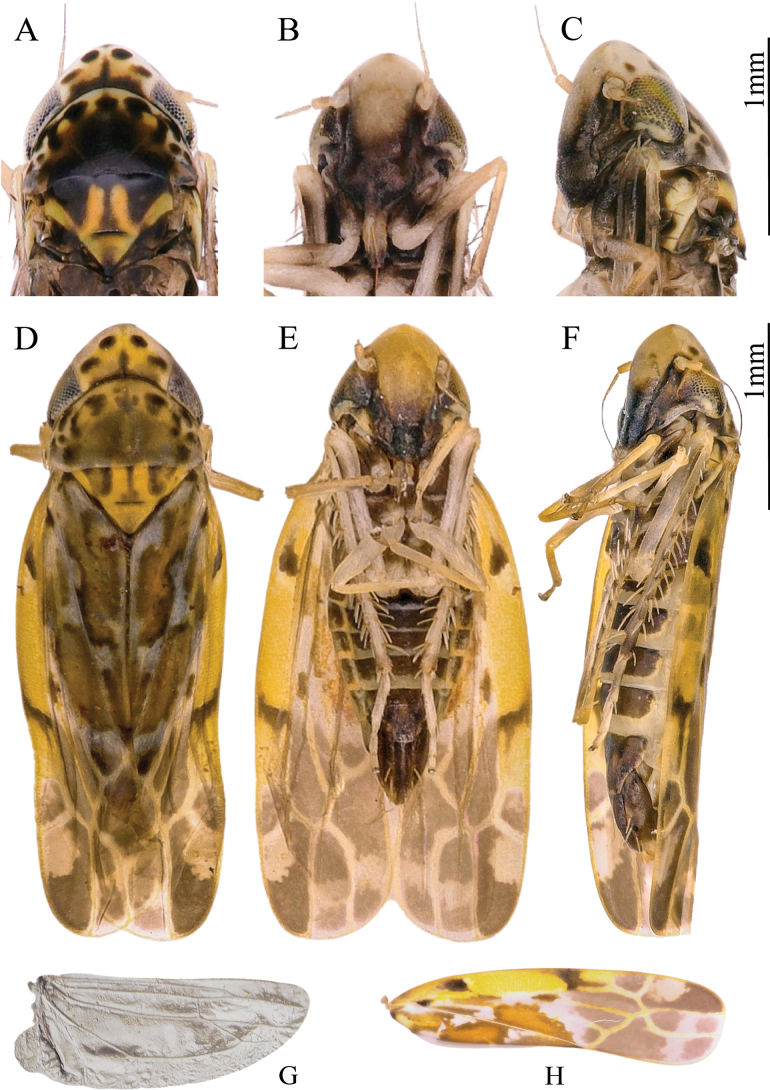
*Thapaia malingensis* Yuan & Song, sp. nov. A. Head and thorax, dorsal view; B. Face, ventral view; C. Face, lateral view; D. Habitus, dorsal view; E. Habitus, ventral view; F. Habitus, lateral view; G. Hind wing; H. Forewing.

**Figure 2.**
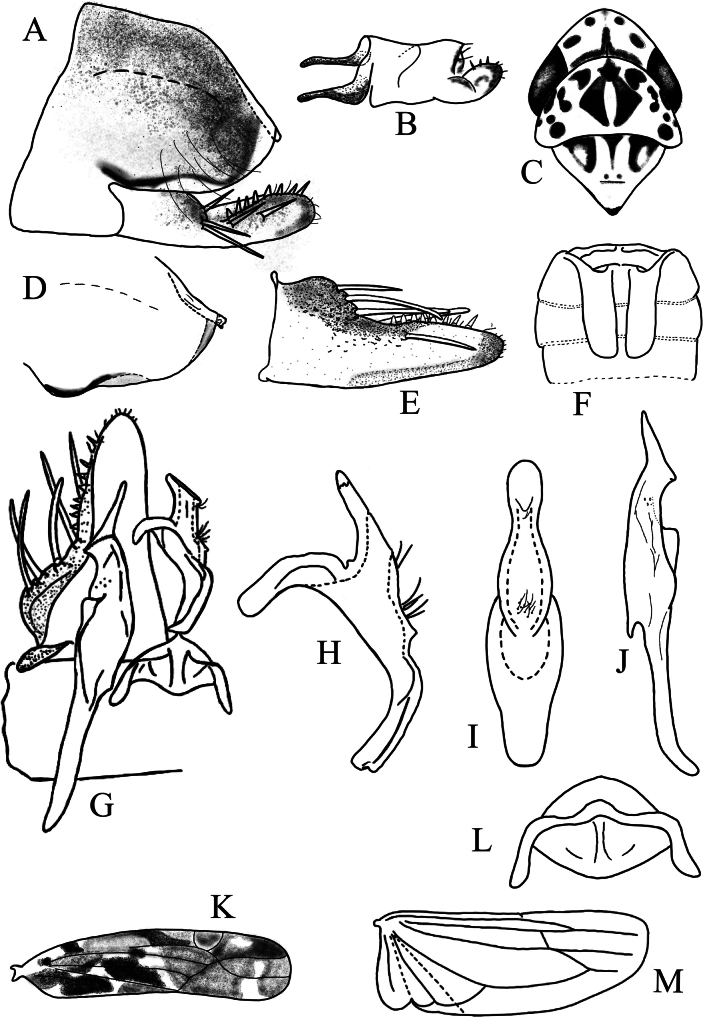
*Thapaia malingensis* Yuan & Song, sp. nov. A. Pygofer, lateral view; B. Anal tube and anal processes, lateral view; C. Head and thorax, dorsal view; D. Apex of pygofer lobe and dorsal pygofer appendage, lateral view; E. Subgenital plate; F. Abdominal apodemes; G. Connective, style, subgenital plate and aedeagal shaft; H. Aedeagus, lateral view; I. Aedeagus, ventral view; J. Style; K. Forewing; L. Connective; M. Hind wing.

Body light brown. Vertex and pronotum light brownish yellow (Fig. 1A, D). Vertex anterior margin protruding medially, with 4 dark brown spots (Figs 1A, D, 2C). Coronal suture distinct, with dark brown patch along midline and vertex–pronotum junction. Face brownish yellow, with dark brown anteclypeus (Fig. 1B, E). Pronotum yellowish, with large, dark-brown to black markings. Scutellum yellow or orange (Figs 1A, D, 2C). Forewing brownish white, with numerous large brown or orangish-brown markings; brochosome brightly ochre-yellow; 1^st^ apical cell with oblique base, 2^nd^ apical cell petiolate basally, 4^th^ apical cell very small (Fig. 2K). Hind wing without RA (Fig. 2M).

Abdominal apodemes broad, extending to middle area of 5^th^ sternite (Fig. 2F).

***Male genitalia*.** Pygofer (Fig. 2A) broad, with long, fine setae near caudo-ventral margin; lobe near tail tip almost transparent; dorsal pygofer appendage fused to lobe (Fig. 2D). Anal tube with pair of basal processes (Fig. 2B). Subgenital plate (Fig. 2E) with base twice as wide as apex, numerous stout setae from middle to tip, and five macrosetae at concave area subbasally. Style (Fig. 2J) slender, apically elongate. Aedeagal shaft (Fig. 2G−I) curved dorsally in lateral view, several short, fine setae located at base of shaft and separately near gonopore; preatrium as long as dorsal apodeme. Connective (Fig. 2L) with lateral arms long and central lobe large.

#### Measurements.

Body length in males 3.3–3.5 mm, in females 3.2–3.5 mm.

#### Material examined.

***Holotype*** male: China, • Guizhou Prov. Maling River Canyon, 700–800 m, 25.1345°N, 104.9733°E, 26 Jan. 2025, coll. Wenming Xu (specimen no. MLH2X-1; deposited in the School of Karst Science, Guizhou Normal University, Guiyang, China [GZNU]). ***Paratypes***: • one male, seven females, same data as holotype (specimen no. MLH2X-2, MLH2C-1−MLH2C-7; deposited as above).

#### Remarks.

The new species can be distinguished from other species by the following characteristics: aedeagal shaft without any processes (Fig. 2G−I); abdominal apodemes extending to 5^th^ sternite (Fig. 2F); subgenital plate with 5 macrosetae subbasally at concave aera (Fig. 2E); and style with pointed apex and a secondary dentate-like extension (Fig. 2J).

#### Etymology.

The specific name refers to its type locality from Maling River Canyon, Guizhou.

## Supplementary Material

XML Treatment for Thapaia

XML Treatment for Thapaia malingensis

## ﻿Additional information

### Use of AI

No use of AI was reported.

### Author ORCIDs

Xiaojuan Yuan https://orcid.org/0009-0006-0743-2019

Wenming Xu https://orcid.org/0009-0004-8936-1650

Zihui Zhao https://orcid.org/0009-0008-1130-2553

Di Su https://orcid.org/0009-0003-6701-0836

Yuehua Song https://orcid.org/0000-0003-3567-3056

### Data availability

All of the data that support the findings of this study are available in the main text.
